# Expression of Placental Neurotrophin-3 (NT-3) in Physiological Pregnancy, Preeclampsia and Chorioamnionitis

**DOI:** 10.4137/cpath.s2325

**Published:** 2009-06-11

**Authors:** Alessandra Casciaro, Felice Arcuri, Rossella Occhini, M. Stefania Toti, Claudio De Felice, Paolo Toti

**Affiliations:** 1 Department of Human Pathology and Oncology; 2 Department of Obstetrics, Gynecology and Reproductive Medicine, University of Siena, 53100 Siena, Italy

**Keywords:** neurotrophins, pregnancy, placenta, chorioamnionitis, preeclampsia

## Abstract

Neurotrophic factors are a group of proteins that act as paracrine and autocrine growth factors. They are involved in the regulation of morphogenesis and development of several tissues. The present study aims to evaluate, for the first time, the expression of Neurotrophin-3 in the human placenta during normal pregnancy and in preeclampsia and chorioamnionitis. Neurotrophin-3 mRNA, assessed by RT-PCR analysis in six term placentas, were observed in all the specimens examined. Neurotrophin-3 protein expression and tissue distribution was evaluated by immunohistochemistry in placenta samples from uncomplicated first trimester (n = 5) and term (n = 5) pregnancies as well as in specimens from preeclampsia (n = 5) and chorioamnionitis (n = 5). In first trimester specimens, strong immunoreactivity was present in villous stromal cells, in the cyto- and syncytiotrophoblast, in decidua cells and in endometrial glands. Third trimester specimens showed prominent immunostaining in cyto- and syncytiotrophoblast cells, in decidua cells and in the amniotic membranes. Villous stromal cells were weakly stained. Similar protein localization was observed in placentas with preeclampsia and chorioamnionitis. In the latter, however, positive villous stromal cells increased in number and in staining intensity when compared with controls and preeclampsia (p < 0.001). The roles of Neurotrophin-3 in pregnancy are presently unknown. A regulatory function on placenta and foetal brain development and maternal inflammatory response may be hypothesized.

## Introduction

Neurotrophins (NT) are a group of functionally and structurally related polypeptides that share about 50% amino acid sequence and act as paracrine and autocrine growth factors. One gene family encodes the neurotrophins, and amino acids are clustered in conserved regions alternated by variable regions; this combination defines the biological functions of these proteins.^1^ Neurotrophic factors comprise a family of proteins including nerve growth factor (NGF), brain-derived neurotrophic factor (BDNF), neurotrophin 3 (NT-3) and neurotrophin 4 (NT-4). These proteins control generation, survival, differentiation and death of neurons in the peripheral and central nervous system in embryonic and postnatal stages, as well as neuronal maintenance later in life.^2^,^3^ Outside the nervous system, NTs can regulate morphogenesis, proliferation and apoptosis^4^–^6^ and seem to be involved in the development of organs such as kidney, muscles and heart.^7^,^8^

The NT-3 gene is localized on chromosome 12^9^ and it is considered the neurotrophin ancestor gene.^9^,^10^ NT-3 presents a structure similar to that of NGF and BDNF,^5^,^11^,^12^ but unlike them, its levels in the central nervous system are higher during foetal development than in adult life.^6^,^11^ In particular, NT-3 is thought to be implicated in the development and maturation of sympathetic neurons. In migratory neural crest cells, the reported mitotic effect of NT-3 indicates an involvement in gangliogenesis and highlights an autocrine and/or paracrine action required for proliferation, differentiation and survival of sympathetic neuroblasts.^13^

We have previously demonstrated the expression of NGF mRNA and peptide in human trophoblast, decidua and fetal membranes in first and third trimester of pregnancy, hypothesizing a role for this neurotrophic factor in the regulation of placental development and in fetal growth.^14^ The aim of the present study is to examine the expression of a different neurotrophic factor, NT-3, in the human placenta. In the view of the numerous different actions of neurotrophins, we have also investigated the expression of NT3 in placental samples from women with chorioamnionitis and preeclampsia, the most frequent pathologies of the third trimester of pregnancy.

## Materials and Methods

### Tissue collection

Specimens of human placenta and decidua were obtained from five pregnancies electively terminated between 8 and 12 weeks of gestation, and from five pregnancies at term (gestational age between 38 and 40 weeks) after elective cesarean section. Gestational age was calculated on the basis of the last menstrual period and confirmed by ultrasound evaluation. Specimens were also obtained from preeclamptic women (n = 5) (low or mild form of the disease, elective cesarean section between 37 and 39 weeks) and from patients with chorioamnionitis (n = 5) (cesarean section between 37 and 40 weeks). The diagnosis of preeclampsia was made on clinical evidence of maternal hypertension and increased urinary proteins.^15^ The diagnosis of subclinical, histologic chorioamnionitis was made at histological examination by the presence of acute inflammatory infiltrate in the amniotic membranes and umbilical cords.^16^ Three patients presented with antecedent PPROM. The study was approved by the local ethics committee (University of Siena) and informed consent was obtained from all women. The specimens were separated in different aliquots: i) immediately submerged in a RNA stabilization reagent (RNAlater; QIAGEN, Valencia, CA, USA) for subsequent extraction of total RNA and reverse transcriptase-polymerase chain reaction (RT-PCR) and; ii) fixed by immersion in 10% buffered formalin for the evaluation of peptide localization by immunohistochemistry. Placenta sampling for histology included multiple full-thickness placental blocks (10 × 10 mm), membranes, and sections of the umbilical cord at three different levels. For each specimen, a block representing both the maternal decidua and the amnion layer was selected for IHC.

### RNA extraction and RT-PCR

Total RNA was extracted from normal placenta at term (n = 6) using the SV total RNA extraction kit (Promega Corporation Madison, WI, USA) following the procedure indicated by the manufacturer. RNA integrity was tested by agarose gel electrophoresis in the presence of 2.2 M formaldehyde. NT-3 mRNA was detected by reverse transcriptase-polymerase chain reaction (RT-PCR). One μg of total RNA was diluted in a reaction buffer volume of 20 μL. The mixture was incubated at 42 °C for 15 minutes, 99 °C for 5 minutes and 5 °C for 5 minutes in a programmable thermal cycler (Bio-Rad, Hercules, CA, USA). For each RNA specimen, a negative control was prepared by omitting the reverse transcriptase. Gene-specific primers used for PCR were chosen according to the published sequence of the human NT-3 and GAPDH mRNA (GenBank NM002527 and NM 002046). The NT-3 forward primer was 5′-TGGCATCCAAGGTAACAACA-3′, the reverse primer was 5′-CTCTGTTGTCGCAGCAGTTC-3′, and expected size of the amplified fragment was 249 bp. The GAPDH forward primer was 5′-GAAGGTGAAGGTCGGAGTC-3′, the reverse primer was 5′-GAAGATGGTGATGGGATTTC-3′ and the expected size of the amplified fragment was 226 bp. For RT-PCR, 2 μL of RT reaction product was added to a mix containing 5X reaction buffer [300 mM Tris-HCl (pH 8.5), 75 mM (NH_4_)_2_SO_4_, 7.5 mM MgCl_2_], dNTP mixture (final concentration 0.25 mM), 1.0 U cloned *Thermus aquaticus* DNA polymerase (Invitrogen Corporation Carlsbad, CA, USA), and NT-3 primers (final concentration 0.4 μL) in a volume of 50 μL. PCR was carried out for 1 minute at 94 °C, 1 minute at 60 °C and 1 minute at 72 °C for 35 cycles followed by a final 10 minutes at 72 °C. For each specimen, a blank was prepared using 2 μL of the corresponding RT blank. One-fifth of each PCR solution was fractioned by electrophoresis in a 1.8% agarose gel. Gels were stained with ethidium bromide, destained and photographed.

### Immunohistochemistry

To evaluate the localization of NT-3, immunohistochemistry was carried out on 5 μm thick sections obtained from paraffin-embedded samples, mounted on electrostatically charged slides and dried overnight at 37 °C. Sections were de-waxed, re-hydrated and washed in Tris-buffered saline [TBS: 20 mM Tris-HCl, 150 mM NaCl (pH 7.6)]. Tissues were rinsed in 3% hydrogen peroxide to block endogenous peroxidase and heated in a microwave oven 3 times for 5 minutes at 750 W in EDTA solution (0.05 M pH 8). Slides were incubated 1 hour at room temperature with primary antibody. NT-3 polyclonal goat antibody was obtained from R&D systems (Minneapolis, MN, USA) and used at the dilution of 1:300. The reaction was developed by the streptavidin-biotin technique (Universal DakoCytomation LSAB^®^ + Kit, Peroxidase; Thermo Fisher Scientific, Fremont, CA, USA) using diaminobenzidine tetrahydrochloride as chromogen (DakoCytomation Liquid DAB substrate Chromogen System). Harris hematoxylin was used for nuclear counterstaining. A positive reaction was characterized by the presence of granular brown staining in the cytoplasm.

The intensity of immunostaining was graduated in a semiquantitative manner. In particular, 0 indicated absence of stain, + indicated weak staining in few cells (below 10%), ++ indicated positivity in several cells (between 10 and 80%), +++ indicates strong positivity in most of the cells (over 80%).

### Statistics

The intensity of staining in control versus chorioamnionitis or preeclampsia cases was compared using the chi-square test, with significance set at a probability value of *p* < 0.05.

## Results

### RT-PCR

**Figure 1.**
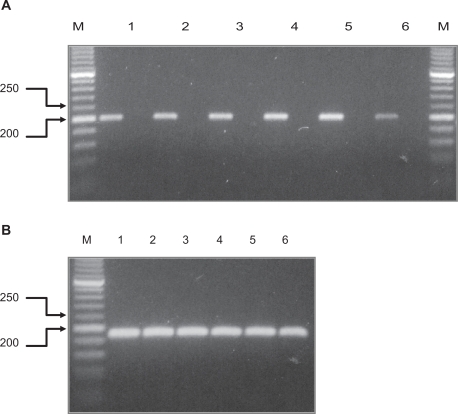
Reverse transcriptase—PCR analysis of NT-3 mRNA levels (**A**) in placental tissues. One μg of total RNA of six placenta specimens (lanes 1–6) was reverse transcribed and amplified in the presence of NT-3 (A) and Glyceraldehyde 3 phosphate dehydrogenase (GAPDH) (**B**) primers. For each specimen a negative control, prepared by omitting the reverse transcriptase, was amplified and loaded onto the gel (A: unmarked lanes). Thirty cycles were run for each PCR. The size of the molecular weight makers (lanes M; bp) is indicated.

The expression of NT-3 in human placenta was first evaluated by RT-PCR analysis. One μg of total RNA extracted from term placenta (n = 6) was reverse transcribed and amplified in the presence of NT-3 primers. An intense band, corresponding in size to the NT-3 product, was obtained from the cDNA of all the specimens examined (Fig. 1). The identity of the NT-3 cDNA was confirmed by direct DNA sequencing. Lack of PCR products was observed in negative controls obtained by omitting the reverse transcriptase (Fig. 1).

### Immunohistochemistry

**Figure 2.**
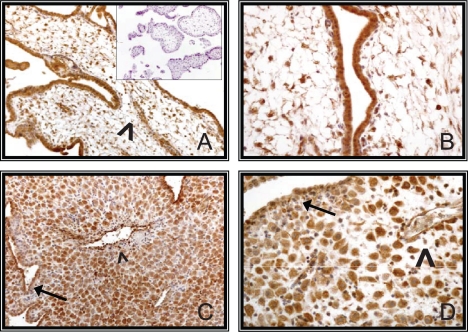
Immunohistochemical analysis of NT-3 expression in first trimester samples. A (100X, original magnification) immature villi (insert, negative control); arrowhead indicates a fetal vessel. B (400X), immature villi. C (100X) and D (400X), decidua; arrows indicate endometrial glands; arrowheads show maternal vessels.

Immunostaining of paraffin-embedded sections of human placenta revealed that NT-3 was strongly expressed throughout pregnancy. In first trimester placenta, strong and consistent immunopositivity was evident in villous stromal cells and in the cyto- and syncytiotrophoblast. Fetal vessels were also intensively stained (Fig. 2A and 2B). On the maternal side, positivity was observed in the decidualized stromal cells and in the epithelial cells of endometrial glands. Walls of maternal vessels were also intensively stained (Fig. 2C and 2D).

**Figure 3.**
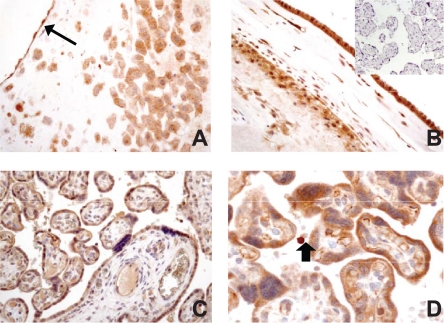
Immunohistochemical analysis of NT-3 expression in normal third trimester samples. **A** (200X, original magnification) decidua (arrow indicates a maternal vessel lined by extravillous trophoblast). **B** (200X), amniotic membranes (insert, negative control). **C** (100X) and **D** (400X) mature villi (D: arrow indicates a maternal macrophage).

In third trimester specimens from physiological pregnancies, maternal decidua cells showed an intense positivity. Extravillous trophoblast lining maternal decidual vessels also expressed NT-3 (Fig. 3A). Fetal membranes presented immunopositivity in amniotic cells, in the stroma cells of the chorionic layer, in the extravillous trophoblast and in the decidua capsularis (Fig. 3B). Normal term placenta presented strong protein expression in villous cytotrophoblast and syncytiotrophoblast. However, unlike first trimester placenta, only a minority of villous stromal cells were stained. Fetal vessel walls were positively stained (Fig. 3C and 3D).

**Figure 4.**
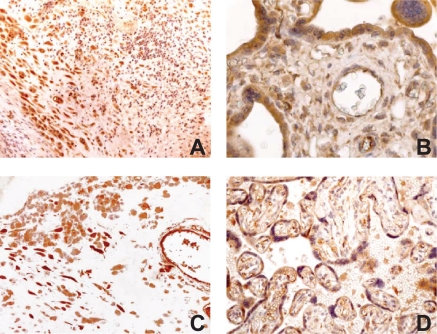
Immunohistochemical analysis of NT-3 expression in chorioamnionitis (A, 100X and B, 400X) and preeclampsia (C and D, 200X). In B all the cells present in the villous stroma are positively immunostained.

In chorioamnionitis, the number and the intensity of staining of villous stromal cells increased compared to normal control tissues (Fig. 4B). Statistical analysis confirmed an increased expression of NT3 in the stroma of the terminal villi, either when comparing chorioamnionitis to normal pregnancy (Table) or to preeclampsia (data not shown) (chi-square test, p < 0.001, DF = 3). Cyto- and syncytiotrophoblast, fetal and maternal vessel cells and decidua showed a staining intensity comparable to that of control tissues (Fig. 4A and 4B).

No staining differences were observed in specimens with preeclampsia compared to control tissues. Immunopositivity was localized in the cyto- and syncytiotrophoblast cells and in the endothelial cells of fetal vessels. In the villous stroma only few cells were stained. Decidua was also stained together with some sporadic inflammatory cells (Fig. 4C and 4D).

## Discussion

The present study demonstrated, for the first time, the presence of NT-3 transcript and protein in the physiological and pathological placenta throughout pregnancy. The exact role and functions of placental neurotrophins are presently unknown. Previous studies, however, have established that these proteins play a key-role in the developing central and peripheral nervous system and that they might also modulate angiogenesis and affect the development of several organs and tissues.^10^ These data might therefore suggest a role for neurotrophins in the developing placenta and growing foetus. Indeed, as described by Malamitsi-Purcher et al^17^ high levels of circulating neurotrophins are found in both foetal and maternal plasma. These authors proposed that circulating levels of neurotrophins mirror the central nervous system levels, since these proteins can easily cross the immature blood-brain barrier. Yet, maternal plasma levels of neurotrophins are much higher than fetal/neonatal levels.^18^ The results of the present study, showing NT-3 expression by decidua and trophoblast cells lining the maternal blood spaces in the placenta, indicate these cells as a likely source of circulating NT-3 in the maternal plasma.

Neurotrophin signal transduction cascade is initiated by the interaction with specific high affinity receptors, the tropomyosin-related tyrosine kinase receptors A-C (TrkA for NGF, TrkB for BDNF and NT4, and TrkC for NT3) and with the low affinity receptor p75^NTR.19–23^ These receptors are expressed in neurons of central and peripheral nervous system, although the Trk and the p75^NTR^ receptors are also found in non-neuronal cells including immune cells, smooth muscle and epithelial cells and fibroblasts.^7^ Immune cells, including B and T lymphocytes and monocytes,^24^ can synthesize and secrete a variety of neurotrophins, including NGF, BDNF, NT-3 and NT-4 and are found to express neurotrophin receptors such as TrkA, TrkB and TrkC. These are believed to be activated in an autocrine and paracrine fashion.^25^ Moreover, it has been observed that antigen activation increases neurotrophin secretion by lymphocytes.^26^ Thus, immune cells express neurotrophin receptors and can be targeted through an autocrine and/or paracrine mechanism.

An additional observation of the present study is the increased NT-3 expression in placental fetal tissues during acute inflammation. We speculate that over expression of NT-3 may influence and regulate inflammatory cell migration to the placenta and fetal membranes, as has been described in different contests.^27^ Indeed, TrkC, the high affinity receptor for NT-3, is expressed by human monocytes, T-lymphocytes and mast cells.^28^–^31^ Through this pathway, NT-3 could modulate the production of granulocytes chemoattractant, such as interleukin-1β, tumor necrosis factor α, and colony stimulating factors. Furthermore, augmented placental production of NT-3 might also increase the circulating levels of the protein in the fetal plasma. Thus, placenta neurotrophin could play a role in protecting the developing fetal brain by damages induced directly or indirectly by microorganisms and by the placental inflammatory reaction.

In conclusion, the present study showed that placenta and fetal membranes produce NT-3. Neurotrophin expression may affect placenta and foetal brain development and maternal inflammatory response. Additional studies will help to better clarify the exact role that these proteins play in the physiopathology of human pregnancy.

## Figures and Tables

**Table 1.** Immunostaining of NT-3 in physiological pregnancy and chorioamnionitis.

	**0**	**+**	**++**	**+++**
Physiological pregnancy	n = 0	n = 4	n = 1	n = 0
Chorioamnionitis	n = 0	n = 0	n = 1	n = 4

Five specimens of physiological pregnancy and five chorioamnionitis were examined. The following scoring system was used: 0, absence of stain; +, weak staining in few cells (below 10%); ++, positivity in several cells (between 10 and 80%); +++, strong positivity in most of the cells (over 80%).
